# Supplementary material for: Aging Leads to Altered Physiological Reactivity in Response to Repeated Social Separation Stress in a Nonhuman Primate Model
Source: bioRxiv. 2025 Oct 6:2025.10.03.680353. Preprint. [Version 1] doi: 10.1101/2025.10.03.680353 (PMC12632632; doi:10.1101/2025.10.03.680353)
Supplement: Supplement 1 [file media-1.pdf]

SUPPLEMENTAL MATERIALS

Aging Leads to Altered Physiological Reactivity in Response to Repeated Social Separation Stress in a Nonhuman Primate Model

Aaryn Mustoe[1*], Jessica Greig[1], Addaline Alvarez[1], Clarissa Hinojosa[1], Jessica Duran[1], Alanna Melchor[1], Eden Comer[1], Juan-Pablo Arroyo[1], Hillary F. Huber[1], Donna Layne-Colon[1], Ektoras Lambrou[2], Jessica Callery[2], Luis Giavedoni[2], Kimberley A. Phillips[1,3], Emily S. Rothwell[4], Adam B. Salmon[5,6], Corinna N. Ross[1]

[1]Southwest National Primate Research Center, Texas Biomedical Research Institute, San Antonio, TX, USA
[2]Department of Biology, Trinity University, San Antonio, TX, USA
[3]Department of Psychology, Trinity University, San Antonio, TX, USA
[4]Department of Neurobiology, University of Pittsburgh School of Medicine, Pittsburgh, PA, USA
[5]Barshop Institute for Longevity and Aging Studies and Department of Molecular Medicine, University of Texas Health San Antonio, San Antonio, TX, USA
[6]Geriatric Research Education and Clinical Center, South Texas Veterans Healthcare Center, San Antonio, TX, USA

*Corresponding Author:
Aaryn Mustoe
Southwest National Primate Research Center
Texas Biomedical Research Institute
amustoe@txbiomed.org

TABLE OF CONTENTS

SI Figure 1. Plots of average cortisol ± SEM across all ten SSCs for each individual marmoset subjects
SI Figure 2. Correlation heatmap for HPA and behavioral measures during the first and last three SSCs
SI Figure 3. Correlation heatmap for HPA and social reunion behavior for all SSCs
SI Figure 4. Correlation heatmap for Blood Chem/CBC parameters.
SI Figure 5. PCA Biplot from HPA and social reunion behavior parameters for reactor and non-reactor
SI Table 1. T-Tests for each measured parameter based on age group
SI Table 2. T-Tests for each measured parameter based on sex
SI Table 3. T-Tests for each measured parameter based on reactor group
SI File 1. Excel tables of "raw" HPA and behavior data and data averaged across all 10 SSCs.

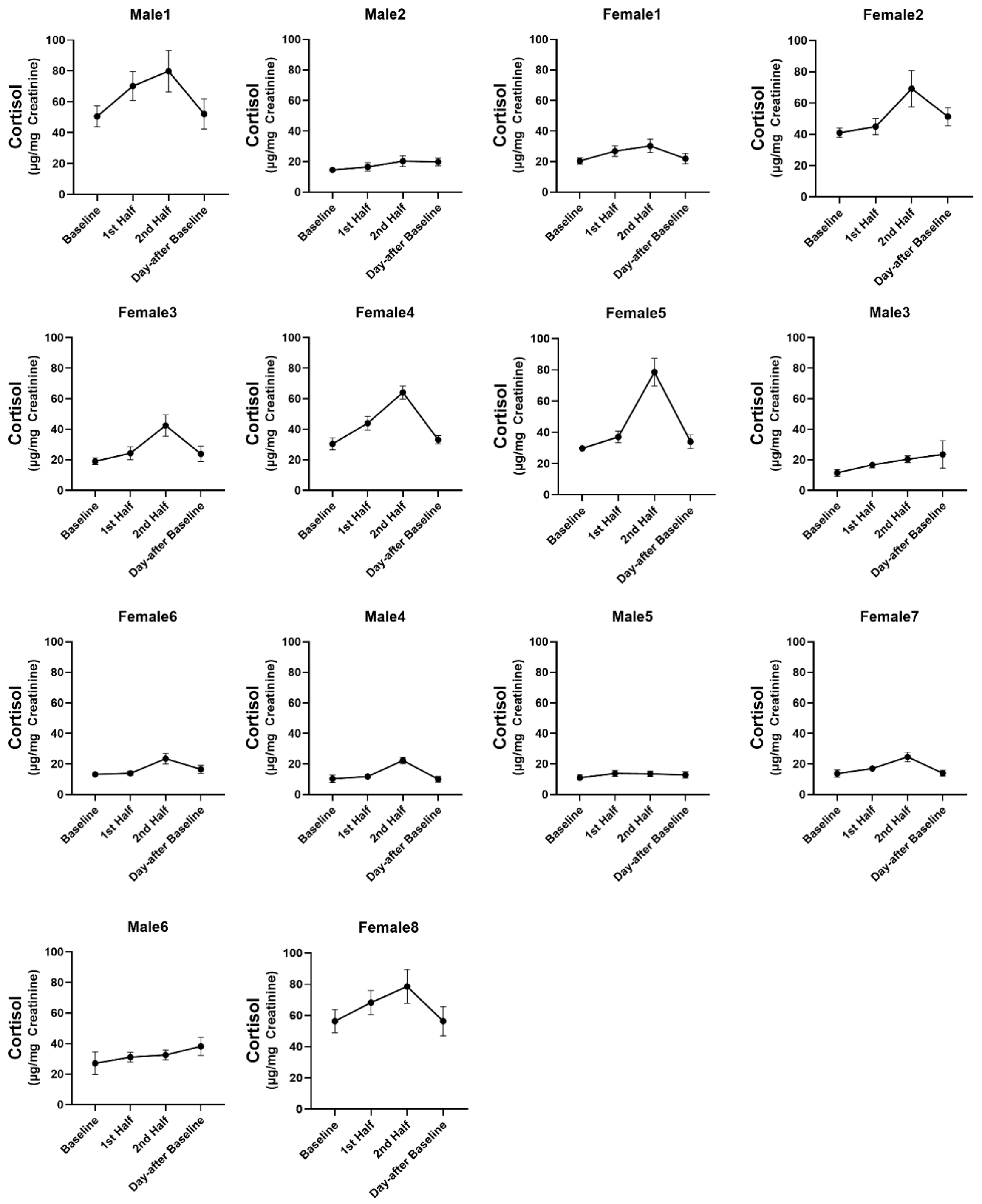

Figure S1. Individual plots of cortisol concentration (mean +/- SEM) averaged across all 10 SSC sessions for each individual participant.

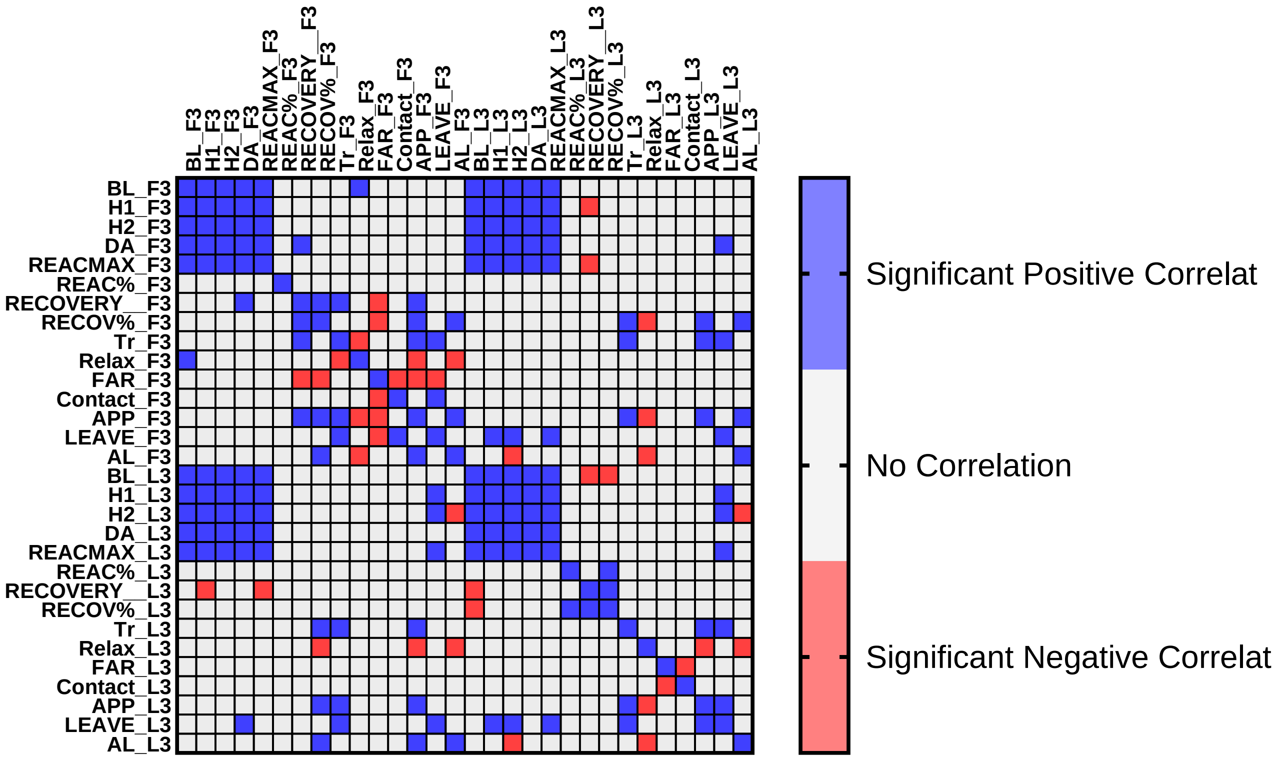

Figure S2. Correlation heat map for representative HPA and social reunion parameters during the first three SSCs (F3) and last three SSCs (L3). APP = approaches, AL = approach to leave ratio. BL = baseline cortisol; DA = day-after baseline cortisol.

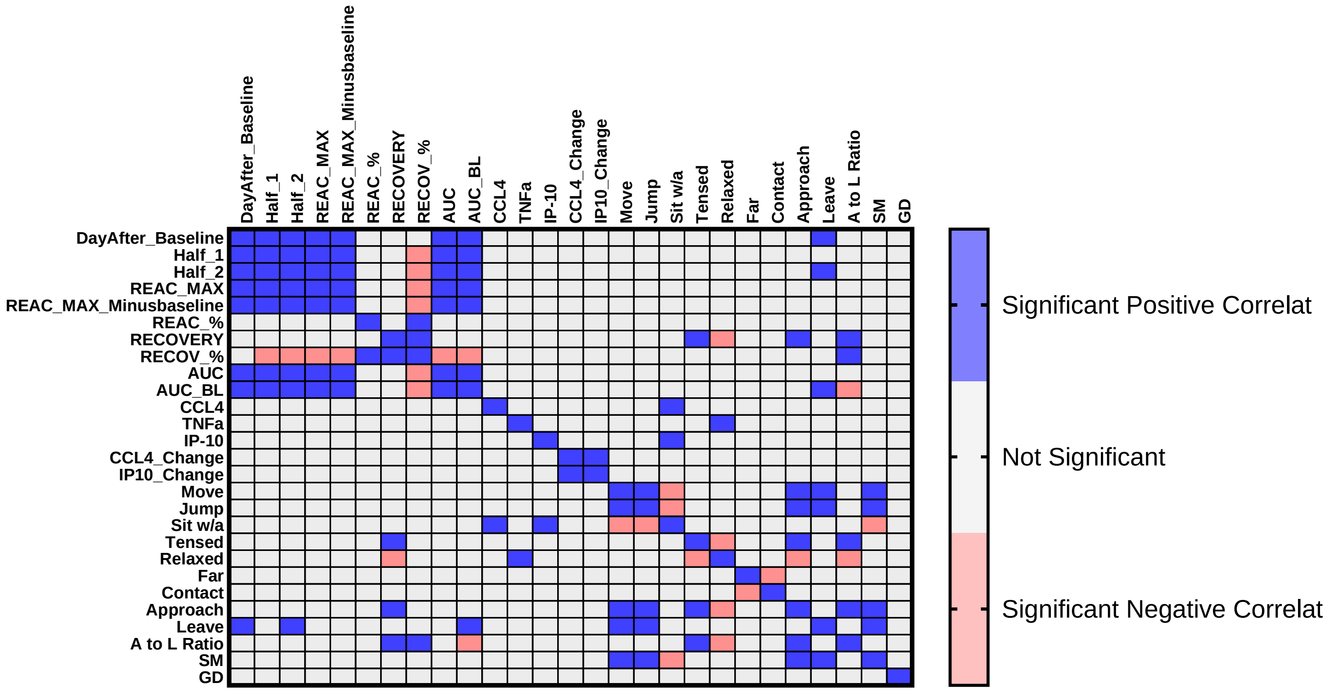

Figure S2. Correlation heat map for representative HPA and social reunion parameters across all 10 SSCs.

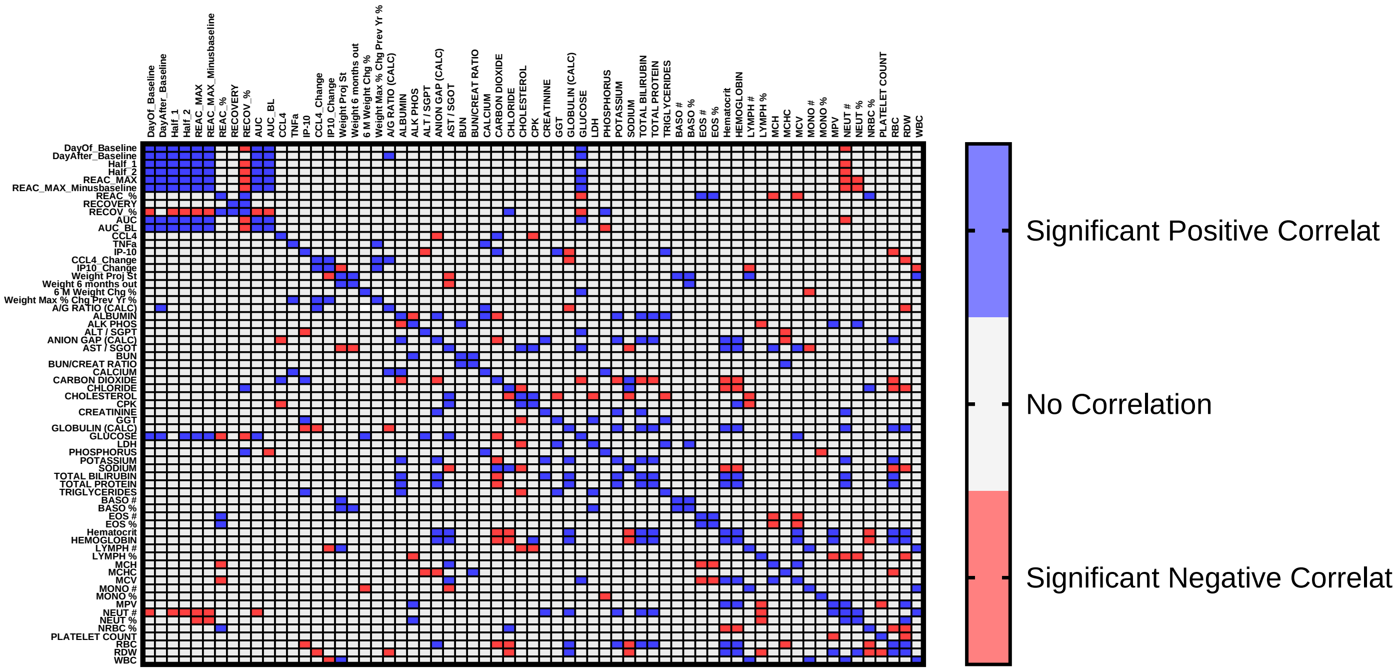

Figure S3. Correlation heat map for HPA and blood CBC/chemistry parameters.

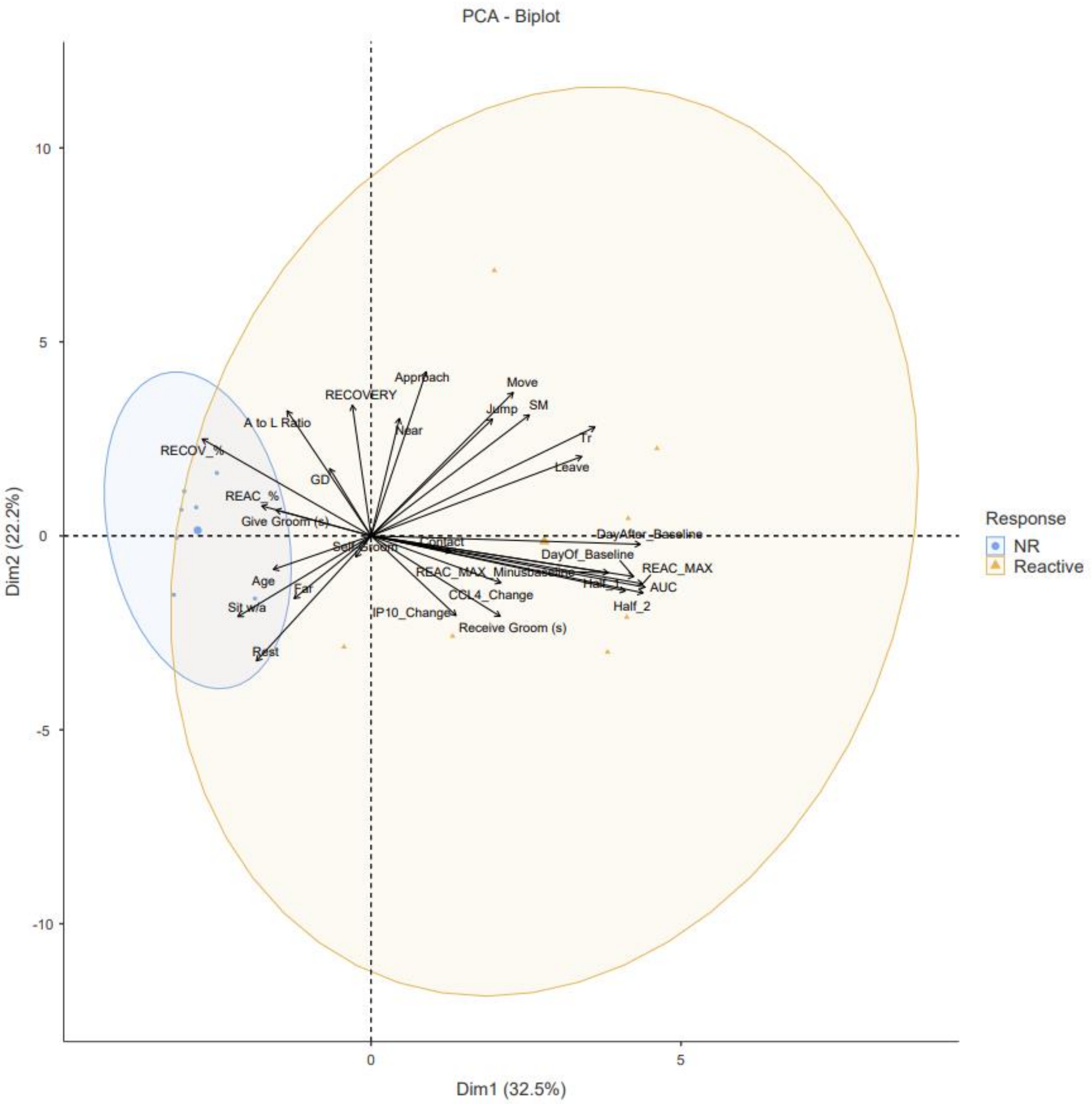

Figure S4. PCA biplot figure of social reunion behavior and cytokine data grouped by individuals who were reactor (orange) and non-reactor (blue) in their HPA responses.

Independent Samples T-Test by Age

		Statistic	df	p		Effect Size
Pair_Length	Student's t	-0.81236	12.0	0.432	Cohen's d	-0.43423
Previous Partners (PP)	Student's t	-0.34133	12.0	0.739	Cohen's d	-0.18245
DayOf_Baseline	Student's t	0.44711	12.0	0.663	Cohen's d	0.23899
8	Student's t	0.22736	12.0	0.824	Cohen's d	0.12153
9	Student's t	0.62481	12.0	0.544	Cohen's d	0.33397
10	Student's t	0.79424	12.0	0.442	Cohen's d	0.42454
11	Student's t	0.73962	12.0	0.474	Cohen's d	0.39535
DayAfter_Baseline	Student's t	0.38659	12.0	0.706	Cohen's d	0.20664
Half_1	Student's t	0.39127	12.0	0.702	Cohen's d	0.20914
Half_2	Student's t	0.80743	12.0	0.435	Cohen's d	0.43159
REAC_MAX	Student's t	0.62759	12.0	0.542	Cohen's d	0.33546
REAC_MAX_Minusbaseline	Student's t	0.71430	12.0	0.489	Cohen's d	0.38181
REAC_%	Student's t	0.39282	12.0	0.701	Cohen's d	0.20997
CORT_ACCUM	Student's t	0.64896	12.0	0.529	Cohen's d	0.34689
RECOVERY	Student's t	-0.20221	12.0	0.843	Cohen's d	-0.10808
RECOV_%	Student's t	-0.01495	12.0	0.988	Cohen's d	-0.00799
AUC	Student's t	0.61205	12.0	0.552	Cohen's d	0.32716
AUC_BL	Student's t	0.82967	12.0	0.423	Cohen's d	0.44348
CCL4	Student's t	-0.66749	12.0	0.517	Cohen's d	-0.35679
TNFa	Student's t	0.65542[a]	12.0	0.525	Cohen's d	0.35034
IP-10	Student's t	0.34208	12.0	0.738	Cohen's d	0.18285
CCL4_Change	Student's t	1.82305	12.0	0.093	Cohen's d	0.97446
IP10_Change	Student's t	1.87440	12.0	0.085	Cohen's d	1.00191
Rest	Student's t	-0.22566	12.0	0.825	Cohen's d	-0.12062
Move	Student's t	0.52827[a]	12.0	0.607	Cohen's d	0.28237
Eat	Student's t	-0.70829	12.0	0.492	Cohen's d	-0.37860
Jump	Student's t	1.06364[a]	12.0	0.308	Cohen's d	0.56854
Hang	Student's t	-0.51628	12.0	0.615	Cohen's d	-0.27596
Sit w/o	Student's t	1.27499[a]	12.0	0.226	Cohen's d	0.68151
Sit w/a	Student's t	-0.16004	12.0	0.876	Cohen's d	-0.08554
1	Student's t	-0.91408	12.0	0.379	Cohen's d	-0.48859
2	Student's t	-1.31127	12.0	0.214	Cohen's d	-0.70090
3	Student's t	1.66918	12.0	0.121	Cohen's d	0.89222
4	Student's t	0.67937	12.0	0.510	Cohen's d	0.36314
Tr	Student's t	0.34029	12.0	0.740	Cohen's d	0.18189
Tensed	Student's t	-0.61863[a]	11.0	0.549	Cohen's d	-0.34417
Relaxed	Student's t	0.48671	11.0	0.636	Cohen's d	0.27078
OV	Student's t	0.53100	11.0	0.606	Cohen's d	0.29542
Far	Student's t	-1.61475	12.0	0.132	Cohen's d	-0.86312

Independent Samples T-Test by Age

		Statistic	df	p		Effect Size
Near	Student's t	0.81256	12.0	0.432	Cohen's d	0.43433
Contact	Student's t	1.47111	12.0	0.167	Cohen's d	0.78634
Approach	Student's t	0.46389	12.0	0.651	Cohen's d	0.24796
Approach latency (s)	Student's t	0.12708	12.0	0.901	Cohen's d	0.06793
Leave	Student's t	0.78495	12.0	0.448	Cohen's d	0.41957
Leave Latency (s)	Student's t	-0.69801	12.0	0.498	Cohen's d	-0.37310
A to L Ratio	Student's t	-0.54436	12.0	0.596	Cohen's d	-0.29098
Receive Groom	Student's t	0.68192	12.0	0.508	Cohen's d	0.36450
Receive Groom (s)	Student's t	0.24892	12.0	0.808	Cohen's d	0.13306
Give Groom	Student's t	1.06066	12.0	0.310	Cohen's d	0.56695
Give Groom (s)	Student's t	0.85993	12.0	0.407	Cohen's d	0.45965
Mate	Student's t	-0.61803	12.0	0.548	Cohen's d	-0.33035
Self-Groom	Student's t	-0.17486	12.0	0.864	Cohen's d	-0.09347
SM	Student's t	-0.05077	12.0	0.960	Cohen's d	-0.02714
GD	Student's t	0.90527	12.0	0.383	Cohen's d	0.48389
Rest (2)	Student's t	1.30766	12.0	0.215	Cohen's d	0.69898
Move (2)	Student's t	-1.11554	12.0	0.286	Cohen's d	-0.59628
Eat (2)	Student's t	-1.45056	12.0	0.173	Cohen's d	-0.77536
Jump (2)	Student's t	-0.78077	12.0	0.450	Cohen's d	-0.41734
Sit	Student's t	-0.00308	12.0	0.998	Cohen's d	-0.00165
Stand	Student's t	0.18853	12.0	0.854	Cohen's d	0.10077
Tr (2)	Student's t	-0.09861	12.0	0.923	Cohen's d	-0.05271
Tensed (2)	Student's t	0.17289	11.0	0.866	Cohen's d	0.09618
Relaxed (2)	Student's t	-0.14832	11.0	0.885	Cohen's d	-0.08252
Head Scans	Student's t	1.98366	12.0	0.071	Cohen's d	1.06031
SM (2)	Student's t	-1.29345	12.0	0.220	Cohen's d	-0.69138
Self Groom	Student's t	-0.33410	12.0	0.744	Cohen's d	-0.17858
In Bucket	Student's t	0.57095	12.0	0.579	Cohen's d	0.30519
Shrill	Student's t	0.43881	12.0	0.669	Cohen's d	0.23455
BCS	Student's t	0.37354	12.0	0.715	Cohen's d	0.19967
Weight	Student's t	0.53485	12.0	0.603	Cohen's d	0.28589
Weight Proj St	Student's t	-0.09713	12.0	0.924	Cohen's d	-0.05192
Weight 6 months out	Student's t	0.93262	12.0	0.369	Cohen's d	0.49851
6 M Weight Chg %	Student's t	1.81582	12.0	0.094	Cohen's d	0.97060
Weight Max % Chg Prev Yr %	Student's t	2.86371	12.0	0.014	Cohen's d	1.53072
A/G RATIO (CALC)	Student's t	0.31634	12.0	0.757	Cohen's d	0.16909
ALBUMIN	Student's t	0.44620	12.0	0.663	Cohen's d	0.23851
ALK PHOS	Student's t	-0.29807	12.0	0.771	Cohen's d	-0.15933
ALT / SGPT	Student's t	-0.11881	12.0	0.907	Cohen's d	-0.06351
ANION GAP (CALC)	Student's t	-0.38007	12.0	0.711	Cohen's d	-0.20315
AST / SGOT	Student's t	0.04127	12.0	0.968	Cohen's d	0.02206

Independent Samples T-Test by Age

		Statistic	df	p		Effect Size
BUN	Student's t	-0.70605	12.0	0.494	Cohen's d	-0.37740
BUN/CREAT RATIO	Student's t	0.74477	12.0	0.471	Cohen's d	0.39809
CALCIUM	Student's t	-0.06110	12.0	0.952	Cohen's d	-0.03266
CARBON DIOXIDE	Student's t	1.51414	12.0	0.156	Cohen's d	0.80934
CHLORIDE	Student's t	-0.89893	12.0	0.386	Cohen's d	-0.48050
CHOLESTEROL	Student's t	-0.67452	12.0	0.513	Cohen's d	-0.36055
CPK	Student's t	-0.45072	11.0	0.661	Cohen's d	-0.25076
CREATININE	Student's t	-0.75574	12.0	0.464	Cohen's d	-0.40396
GGT	Student's t	-1.75487[a]	11.0	0.107	Cohen's d	-0.97632
GLOBULIN (CALC)	Student's t	0.18162	12.0	0.859	Cohen's d	0.09708
GLUCOSE	Student's t	0.81306	12.0	0.432	Cohen's d	0.43460
LDH	Student's t	-0.17062	11.0	0.868	Cohen's d	-0.09492
PHOSPHORUS	Student's t	-0.29605	12.0	0.772	Cohen's d	-0.15824
POTASSIUM	Student's t	1.19811	12.0	0.254	Cohen's d	0.64042
SODIUM	Student's t	-0.31506	12.0	0.758	Cohen's d	-0.16841
TOTAL BILIRUBIN	Student's t	1.27920[a]	12.0	0.225	Cohen's d	0.68376
TOTAL PROTEIN	Student's t	0.40670	12.0	0.691	Cohen's d	0.21739
TRIGLYCERIDES	Student's t	0.06284	11.0	0.951	Cohen's d	0.03496
BASO #	Student's t	0.61237	12.0	0.552	Cohen's d	0.32733
BASO %	Student's t	1.15214	12.0	0.272	Cohen's d	0.61585
EOS #	Student's t	1.47029	12.0	0.167	Cohen's d	0.78591
EOS %	Student's t	1.47625	12.0	0.166	Cohen's d	0.78909
Hematocrit	Student's t	1.51387	12.0	0.156	Cohen's d	0.80920
HEMOGLOBIN	Student's t	1.61155[a]	12.0	0.133	Cohen's d	0.86141
LYMPH #	Student's t	-0.58519	12.0	0.569	Cohen's d	-0.31280
LYMPH %	Student's t	-0.24082	12.0	0.814	Cohen's d	-0.12872
MCH	Student's t	-2.14394	12.0	0.053	Cohen's d	-1.14599
MCHC	Student's t	-0.22706	12.0	0.824	Cohen's d	-0.12137
MCV	Student's t	-1.49277	12.0	0.161	Cohen's d	-0.79792
MONO #	Student's t	-0.96225	12.0	0.355	Cohen's d	-0.51434
MONO %	Student's t	-0.72824	12.0	0.480	Cohen's d	-0.38926
MPV	Student's t	0.04308	12.0	0.966	Cohen's d	0.02303
NEUT #	Student's t	-0.43697[a]	12.0	0.670	Cohen's d	-0.23357
NEUT %	Student's t	0.17079	12.0	0.867	Cohen's d	0.09129
NRBC %	Student's t	-0.07981	12.0	0.938	Cohen's d	-0.04266
PLATELET COUNT	Student's t	0.40738	12.0	0.691	Cohen's d	0.21775
RBC	Student's t	2.14882[a]	12.0	0.053	Cohen's d	1.14859
RDW	Student's t	0.43823	12.0	0.669	Cohen's d	0.23424
WBC	Student's t	-0.80715	12.0	0.435	Cohen's d	-0.43144
A/G RATIO (CALC) (2)	Student's t	-0.80937	11.0	0.435	Cohen's d	-0.45029
ALBUMIN (2)	Student's t	-0.63776	11.0	0.537	Cohen's d	-0.35482

Independent Samples T-Test by Age

		Statistic	df	p		Effect Size
ALK PHOS (2)	Student's t	0.44809	11.0	0.663	Cohen's d	0.24930
ALT / SGPT (2)	Student's t	-0.66667	11.0	0.519	Cohen's d	-0.37090
ANION GAP (CALC) (2)	Student's t	-0.22253	11.0	0.828	Cohen's d	-0.12380
AST / SGOT (2)	Student's t	-0.30189	11.0	0.768	Cohen's d	-0.16796
BUN (2)	Student's t	-0.43192	11.0	0.674	Cohen's d	-0.24030
BUN/CREAT RATIO (2)	Student's t	-0.10406	11.0	0.919	Cohen's d	-0.05789
CALCIUM (2)	Student's t	-0.80204	11.0	0.440	Cohen's d	-0.44621
CARBON DIOXIDE (2)	Student's t	-0.10519	11.0	0.918	Cohen's d	-0.05852
CHLORIDE (2)	Student's t	-0.96976	11.0	0.353	Cohen's d	-0.53953
CHOLESTEROL (2)	Student's t	1.30499[a]	11.0	0.219	Cohen's d	0.72603
CPK (2)	Student's t	0.48322	11.0	0.638	Cohen's d	0.26884
CREATININE (2)	Student's t	-1.03304	11.0	0.324	Cohen's d	-0.57473
GGT (2)	Student's t	-0.26842	11.0	0.793	Cohen's d	-0.14933
GLOBULIN (CALC) (2)	Student's t	0.02753	11.0	0.979	Cohen's d	0.01532
GLUCOSE (2)	Student's t	0.42439	11.0	0.679	Cohen's d	0.23611
LDH (2)	Student's t	-1.17352	11.0	0.265	Cohen's d	-0.65289
PHOSPHORUS (2)	Student's t	-0.84268	11.0	0.417	Cohen's d	-0.46882
POTASSIUM (2)	Student's t	-0.28647	11.0	0.780	Cohen's d	-0.15938
SODIUM (2)	Student's t	-1.20460	11.0	0.254	Cohen's d	-0.67018
TOTAL BILIRUBIN (2)	Student's t	0.02800	11.0	0.978	Cohen's d	0.01558
TOTAL PROTEIN (2)	Student's t	-0.38801	11.0	0.705	Cohen's d	-0.21587
TRIGLYCERIDES (2)	Student's t	0.24848	11.0	0.808	Cohen's d	0.13824
BASO # (2)	Student's t	-0.59027	11.0	0.567	Cohen's d	-0.32839
BASO % (2)	Student's t	-0.44733	11.0	0.663	Cohen's d	-0.24887
EOS # (2)	Student's t	2.02988	11.0	0.067	Cohen's d	1.12932
EOS % (2)	Student's t	1.60414	11.0	0.137	Cohen's d	0.89246
Hematocrit (2)	Student's t	-0.04870	11.0	0.962	Cohen's d	-0.02709
HEMOGLOBIN (2)	Student's t	-0.02669	11.0	0.979	Cohen's d	-0.01485
LYMPH # (2)	Student's t	-2.04110[a]	11.0	0.066	Cohen's d	-1.13556
LYMPH % (2)	Student's t	-1.34947[a]	11.0	0.204	Cohen's d	-0.75078
MCH (2)	Student's t	-0.49388	11.0	0.631	Cohen's d	-0.27477
MCHC (2)	Student's t	-0.02679	11.0	0.979	Cohen's d	-0.01491
MCV (2)	Student's t	-0.41753	11.0	0.684	Cohen's d	-0.23229
MONO # (2)	Student's t	-0.77352[a]	11.0	0.456	Cohen's d	-0.43034
MONO % (2)	Student's t	0.53977	11.0	0.600	Cohen's d	0.30030
MPV (2)	Student's t	-0.45097	11.0	0.661	Cohen's d	-0.25089
NEUT # (2)	Student's t	-0.13005	11.0	0.899	Cohen's d	-0.07235
NEUT % (2)	Student's t	0.35668	11.0	0.728	Cohen's d	0.19844
NRBC % (2)	Student's t	1.39749	11.0	0.190	Cohen's d	0.77749
PLATELET COUNT (2)	Student's t	1.00934	11.0	0.334	Cohen's d	0.56155
RBC (2)	Student's t	0.18013	11.0	0.860	Cohen's d	0.10022

Independent Samples T-Test by Age

		Statistic	df	p		Effect Size
RDW (2)	Student's t	-0.04123	11.0	0.968	Cohen's d	-0.02294
WBC (2)	Student's t	-1.48989[a]	11.0	0.164	Cohen's d	-0.82890

Note. H_a $\mu_{Peri\text{-}Geri} \neq \mu_{Very\text{-}Geri}$

[a] Levene's test is significant ($p < .05$), suggesting a violation of the assumption of equal variances

SI Table 1. T-Tests for each measured parameter based on age group. Behavioral variables followed by “(2)” are isolation behaviors. Blood CBC/Chem parameters are averaged or parameters followed by “(2)” are change from post-pre. Positive t-statistics indicate peri-geri > very-geri. For clarification on Behavioral variable names, see Table 1.

Independent Samples T-Test by Sex

		Statistic	df	p		Effect Size
Pair_Length	Student's t	-1.6867	12.0	0.117	Cohen's d	-0.91094
Previous Partners (PP)	Student's t	0.6484	12.0	0.529	Cohen's d	0.35020
DayOf_Baseline	Student's t	0.8653	12.0	0.404	Cohen's d	0.46734
8	Student's t	0.4576	12.0	0.655	Cohen's d	0.24715
9	Student's t	1.0659	12.0	0.307	Cohen's d	0.57563
10	Student's t	1.4327	12.0	0.177	Cohen's d	0.77374
11	Student's t	1.6382	12.0	0.127	Cohen's d	0.88475
DayAfter_Baseline	Student's t	0.6210	12.0	0.546	Cohen's d	0.33536
Half_1	Student's t	0.7342	12.0	0.477	Cohen's d	0.39650
Half_2	Student's t	1.5320	12.0	0.151	Cohen's d	0.82738
REAC_MAX	Student's t	1.1906	12.0	0.257	Cohen's d	0.64298
REAC_MAX_Minusbaseline	Student's t	1.1728	12.0	0.264	Cohen's d	0.63340
REAC_%	Student's t	-0.5674	12.0	0.581	Cohen's d	-0.30645
CORT_ACCUM	Student's t	1.1828	12.0	0.260	Cohen's d	0.63879
RECOVERY	Student's t	-1.7496[a]	12.0	0.106	Cohen's d	-0.94489
RECOV_%	Student's t	-2.2466	12.0	0.044	Cohen's d	-1.21329
AUC	Student's t	1.1603	12.0	0.268	Cohen's d	0.62662
AUC_BL	Student's t	1.6508	12.0	0.125	Cohen's d	0.89152
CCL4	Student's t	-0.0556[a]	12.0	0.957	Cohen's d	-0.03003
TNFa	Student's t	0.0432	12.0	0.966	Cohen's d	0.02333
IP-10	Student's t	-0.9526	12.0	0.360	Cohen's d	-0.51448
CCL4_Change	Student's t	0.2293	12.0	0.822	Cohen's d	0.12386
IP10_Change	Student's t	0.1019	12.0	0.920	Cohen's d	0.05505
Rest	Student's t	-0.1029	12.0	0.920	Cohen's d	-0.05556
Move	Student's t	-0.3096	12.0	0.762	Cohen's d	-0.16720
Eat	Student's t	0.9641	12.0	0.354	Cohen's d	0.52065
Jump	Student's t	-0.6598	12.0	0.522	Cohen's d	-0.35634
Hang	Student's t	-0.0885	12.0	0.931	Cohen's d	-0.04778
Sit w/o	Student's t	0.7765	12.0	0.453	Cohen's d	0.41934
Sit w/a	Student's t	0.4219	12.0	0.681	Cohen's d	0.22783
1	Student's t	0.5756	12.0	0.576	Cohen's d	0.31084
2	Student's t	-1.3383	12.0	0.206	Cohen's d	-0.72276
3	Student's t	0.7398	12.0	0.474	Cohen's d	0.39953
4	Student's t	-0.0385[a]	12.0	0.970	Cohen's d	-0.02079
Tr	Student's t	-0.1412	12.0	0.890	Cohen's d	-0.07623
Tensed	Student's t	-1.3488	11.0	0.205	Cohen's d	-0.75039
Relaxed	Student's t	1.2031	11.0	0.254	Cohen's d	0.66935
OV	Student's t	0.7120	11.0	0.491	Cohen's d	0.39613
Far	Student's t	0.7924	12.0	0.444	Cohen's d	0.42792
Near	Student's t	-1.4584	12.0	0.170	Cohen's d	-0.78764

Independent Samples T-Test by Sex

		Statistic	df	p		Effect Size
Contact	Student's t	0.0871	12.0	0.932	Cohen's d	0.04704
Approach	Student's t	-2.2652	12.0	0.043	Cohen's d	-1.22333
Approach latency (s)	Student's t	4.5850[a]	12.0	<.001	Cohen's d	2.47616
Leave	Student's t	0.7614	12.0	0.461	Cohen's d	0.41118
Leave Latency (s)	Student's t	-1.3906	12.0	0.190	Cohen's d	-0.75103
A to L Ratio	Student's t	-5.0360[a]	12.0	<.001	Cohen's d	-2.71975
Receive Groom	Student's t	0.9514	12.0	0.360	Cohen's d	0.51383
Receive Groom (s)	Student's t	0.5779	12.0	0.574	Cohen's d	0.31207
Give Groom	Student's t	-0.6968	12.0	0.499	Cohen's d	-0.37632
Give Groom (s)	Student's t	-0.7094	12.0	0.492	Cohen's d	-0.38312
Mate	Student's t	-1.9478[a]	12.0	0.075	Cohen's d	-1.05194
Self-Groom	Student's t	0.9216[a]	12.0	0.375	Cohen's d	0.49775
SM	Student's t	0.1660	12.0	0.871	Cohen's d	0.08967
GD	Student's t	-1.8771[a]	12.0	0.085	Cohen's d	-1.01376
Rest (2)	Student's t	-1.2615	12.0	0.231	Cohen's d	-0.68131
Move (2)	Student's t	1.7907	12.0	0.099	Cohen's d	0.96710
Eat (2)	Student's t	-1.9981	12.0	0.069	Cohen's d	-1.07909
Jump (2)	Student's t	2.2383	12.0	0.045	Cohen's d	1.20884
Sit	Student's t	-2.8771	12.0	0.014	Cohen's d	-1.55383
Stand	Student's t	2.6562	12.0	0.021	Cohen's d	1.43451
Tr (2)	Student's t	2.7315[a]	12.0	0.018	Cohen's d	1.47517
Tensed (2)	Student's t	1.9577	11.0	0.076	Cohen's d	1.08915
Relaxed (2)	Student's t	-1.9504	11.0	0.077	Cohen's d	-1.08511
Head Scans	Student's t	0.9377	12.0	0.367	Cohen's d	0.50643
SM (2)	Student's t	2.0154[a]	12.0	0.067	Cohen's d	1.08844
Self Groom	Student's t	-0.4575[a]	12.0	0.655	Cohen's d	-0.24709
In Bucket	Student's t	-0.1861	12.0	0.855	Cohen's d	-0.10052
Shrill	Student's t	0.4383	12.0	0.669	Cohen's d	0.23672
BCS	Student's t	0.1073	12.0	0.916	Cohen's d	0.05793
Weight	Student's t	0.5605	12.0	0.585	Cohen's d	0.30271
Weight Proj St	Student's t	0.4852	12.0	0.636	Cohen's d	0.26203
Weight 6 months out	Student's t	1.3841	12.0	0.192	Cohen's d	0.74751
6 M Weight Chg %	Student's t	1.4940	12.0	0.161	Cohen's d	0.80686
Weight Max % Chg Prev Yr %	Student's t	0.1671	12.0	0.870	Cohen's d	0.09024
A/G RATIO (CALC)	Student's t	-0.5391	12.0	0.600	Cohen's d	-0.29112
ALBUMIN	Student's t	-0.3495	12.0	0.733	Cohen's d	-0.18877
ALK PHOS	Student's t	0.2808	12.0	0.784	Cohen's d	0.15168
ALT / SGPT	Student's t	1.6779	12.0	0.119	Cohen's d	0.90615
ANION GAP (CALC)	Student's t	0.5505	12.0	0.592	Cohen's d	0.29731
AST / SGOT	Student's t	-1.0082	12.0	0.333	Cohen's d	-0.54448
BUN	Student's t	0.9677	12.0	0.352	Cohen's d	0.52260

Independent Samples T-Test by Sex

		Statistic	df	p		Effect Size
BUN/CREAT RATIO	Student's t	0.6890	12.0	0.504	Cohen's d	0.37210
CALCIUM	Student's t	-0.0764	12.0	0.940	Cohen's d	-0.04128
CARBON DIOXIDE	Student's t	-0.7448	12.0	0.471	Cohen's d	-0.40226
CHLORIDE	Student's t	-0.4689	12.0	0.648	Cohen's d	-0.25325
CHOLESTEROL	Student's t	-1.6075	12.0	0.134	Cohen's d	-0.86815
CPK	Student's t	-1.0423[a]	11.0	0.320	Cohen's d	-0.59419
CREATININE	Student's t	0.5588	12.0	0.587	Cohen's d	0.30179
GGT	Student's t	-0.4506	11.0	0.661	Cohen's d	-0.25690
GLOBULIN (CALC)	Student's t	0.4309	12.0	0.674	Cohen's d	0.23271
GLUCOSE	Student's t	0.4723	12.0	0.645	Cohen's d	0.25507
LDH	Student's t	-0.5282	11.0	0.608	Cohen's d	-0.30111
PHOSPHORUS	Student's t	0.8187	12.0	0.429	Cohen's d	0.44216
POTASSIUM	Student's t	0.1368	12.0	0.893	Cohen's d	0.07388
SODIUM	Student's t	-0.4878	12.0	0.634	Cohen's d	-0.26347
TOTAL BILIRUBIN	Student's t	-0.2315	12.0	0.821	Cohen's d	-0.12500
TOTAL PROTEIN	Student's t	0.0000	12.0	1.000	Cohen's d	0.00000
TRIGLYCERIDES	Student's t	0.0717[a]	11.0	0.944	Cohen's d	0.04087
BASO #	Student's t	0.3499	12.0	0.732	Cohen's d	0.18898
BASO %	Student's t	-1.1377	12.0	0.277	Cohen's d	-0.61444
EOS #	Student's t	0.1676	12.0	0.870	Cohen's d	0.09054
EOS %	Student's t	0.2322	12.0	0.820	Cohen's d	0.12539
Hematocrit	Student's t	-1.5145	12.0	0.156	Cohen's d	-0.81794
HEMOGLOBIN	Student's t	-1.5287	12.0	0.152	Cohen's d	-0.82562
LYMPH #	Student's t	1.1266	12.0	0.282	Cohen's d	0.60845
LYMPH %	Student's t	1.6891[a]	12.0	0.117	Cohen's d	0.91220
MCH	Student's t	0.8168	12.0	0.430	Cohen's d	0.44112
MCHC	Student's t	0.6453[a]	12.0	0.531	Cohen's d	0.34853
MCV	Student's t	0.3999	12.0	0.696	Cohen's d	0.21595
MONO #	Student's t	1.3833	12.0	0.192	Cohen's d	0.74709
MONO %	Student's t	0.6386	12.0	0.535	Cohen's d	0.34488
MPV	Student's t	-0.1494	12.0	0.884	Cohen's d	-0.08067
NEUT #	Student's t	-1.3165	12.0	0.213	Cohen's d	-0.71100
NEUT %	Student's t	-1.7668[a]	12.0	0.103	Cohen's d	-0.95421
NRBC %	Student's t	0.3457	12.0	0.736	Cohen's d	0.18669
PLATELET COUNT	Student's t	-0.6446	12.0	0.531	Cohen's d	-0.34813
RBC	Student's t	-1.7299	12.0	0.109	Cohen's d	-0.93425
RDW	Student's t	0.9241	12.0	0.374	Cohen's d	0.49908
WBC	Student's t	0.6943	12.0	0.501	Cohen's d	0.37497
A/G RATIO (CALC) (2)	Student's t	-0.0168	11.0	0.987	Cohen's d	-0.00957
ALBUMIN (2)	Student's t	-1.6195	11.0	0.134	Cohen's d	-0.92323
ALK PHOS (2)	Student's t	1.3496	11.0	0.204	Cohen's d	0.76938

Independent Samples T-Test by Sex

		Statistic	df	p		Effect Size
ALT / SGPT (2)	Student's t	1.1255	11.0	0.284	Cohen's d	0.64164
ANION GAP (CALC) (2)	Student's t	-0.5287	11.0	0.608	Cohen's d	-0.30141
AST / SGOT (2)	Student's t	0.9820	11.0	0.347	Cohen's d	0.55982
BUN (2)	Student's t	0.8134[a]	11.0	0.433	Cohen's d	0.46371
BUN/CREAT RATIO (2)	Student's t	0.5220[a]	11.0	0.612	Cohen's d	0.29760
CALCIUM (2)	Student's t	-0.3825	11.0	0.709	Cohen's d	-0.21808
CARBON DIOXIDE (2)	Student's t	0.4346	11.0	0.672	Cohen's d	0.24778
CHLORIDE (2)	Student's t	-0.9621	11.0	0.357	Cohen's d	-0.54846
CHOLESTEROL (2)	Student's t	1.3706[a]	11.0	0.198	Cohen's d	0.78136
CPK (2)	Student's t	-0.2613	11.0	0.799	Cohen's d	-0.14896
CREATININE (2)	Student's t	0.0000	11.0	1.000	Cohen's d	0.00000
GGT (2)	Student's t	-1.2322	11.0	0.244	Cohen's d	-0.70247
GLOBULIN (CALC) (2)	Student's t	-0.9276	11.0	0.374	Cohen's d	-0.52882
GLUCOSE (2)	Student's t	1.7912	11.0	0.101	Cohen's d	1.02112
LDH (2)	Student's t	-0.9284	11.0	0.373	Cohen's d	-0.52926
PHOSPHORUS (2)	Student's t	-1.2794	11.0	0.227	Cohen's d	-0.72938
POTASSIUM (2)	Student's t	-1.8074	11.0	0.098	Cohen's d	-1.03038
SODIUM (2)	Student's t	-0.6124	11.0	0.553	Cohen's d	-0.34913
TOTAL BILIRUBIN (2)	Student's t	-1.0871	11.0	0.300	Cohen's d	-0.61972
TOTAL PROTEIN (2)	Student's t	-1.6631	11.0	0.124	Cohen's d	-0.94814
TRIGLYCERIDES (2)	Student's t	-1.5213	11.0	0.156	Cohen's d	-0.86727
BASO # (2)	Student's t	1.7262	11.0	0.112	Cohen's d	0.98411
BASO % (2)	Student's t	0.7659	11.0	0.460	Cohen's d	0.43665
EOS # (2)	Student's t	-1.9470	11.0	0.078	Cohen's d	-1.10996
EOS % (2)	Student's t	-1.5870	11.0	0.141	Cohen's d	-0.90473
Hematocrit (2)	Student's t	0.1387	11.0	0.892	Cohen's d	0.07909
HEMOGLOBIN (2)	Student's t	0.1155	11.0	0.910	Cohen's d	0.06587
LYMPH # (2)	Student's t	-0.2757	11.0	0.788	Cohen's d	-0.15715
LYMPH % (2)	Student's t	0.3378	11.0	0.742	Cohen's d	0.19256
MCH (2)	Student's t	1.0055	11.0	0.336	Cohen's d	0.57321
MCHC (2)	Student's t	-0.1718	11.0	0.867	Cohen's d	-0.09795
MCV (2)	Student's t	1.1828	11.0	0.262	Cohen's d	0.67429
MONO # (2)	Student's t	-1.7443	11.0	0.109	Cohen's d	-0.99440
MONO % (2)	Student's t	-0.6044	11.0	0.558	Cohen's d	-0.34456
MPV (2)	Student's t	1.2051	11.0	0.253	Cohen's d	0.68703
NEUT # (2)	Student's t	-0.6176	11.0	0.549	Cohen's d	-0.35210
NEUT % (2)	Student's t	-0.6460	11.0	0.532	Cohen's d	-0.36826
NRBC % (2)	Student's t	-1.0070	11.0	0.336	Cohen's d	-0.57409
PLATELET COUNT (2)	Student's t	-1.5168	11.0	0.158	Cohen's d	-0.86473
RBC (2)	Student's t	-0.2464	11.0	0.810	Cohen's d	-0.14048
RDW (2)	Student's t	0.2560	11.0	0.803	Cohen's d	0.14597

Independent Samples T-Test by Sex

		Statistic	df	p		Effect Size
WBC (2)	Student's t	-0.6011	11.0	0.560	Cohen's d	-0.34269

Note. H_a $\mu_{Female} \neq \mu_{Male}$

[a] Levene's test is significant ($p < .05$), suggesting a violation of the assumption of equal variances

SI Table 2. T-Tests for each measured parameter based on sex. Behavioral variables followed by "(2)" are isolation behaviors. Blood CBC/Chem parameters are averaged or parameters followed by "(2)" are change from post-pre. Positive t-statistics indicate females > males. For clarification on Behavioral variable names, see Table 1.

Independent Samples T-Test by HPA Reactor

		Statistic	df	p		Effect Size
Pair_Length	Student's t	-0.16426	12.0	0.872	Cohen's d	-0.08780
Previous Partners (PP)	Student's t	0.34133	12.0	0.739	Cohen's d	0.18245
DayOf_Baseline	Student's t	-4.34432[a]	12.0	<.001	Cohen's d	-2.32213
8	Student's t	-3.79642[a]	12.0	0.003	Cohen's d	-2.02927
9	Student's t	-4.77571[a]	12.0	<.001	Cohen's d	-2.55273
10	Student's t	-5.70321[a]	12.0	<.001	Cohen's d	-3.04849
11	Student's t	-5.84599[a]	12.0	<.001	Cohen's d	-3.12481
DayAfter_Baseline	Student's t	-4.91790[a]	12.0	<.001	Cohen's d	-2.62873
Half_1	Student's t	-4.20688[a]	12.0	0.001	Cohen's d	-2.24867
Half_2	Student's t	-5.59988[a]	12.0	<.001	Cohen's d	-2.99326
REAC_MAX	Student's t	-5.05310[a]	12.0	<.001	Cohen's d	-2.70100
REAC_MAX_Minusbaseline	Student's t	-3.73720[a]	12.0	0.003	Cohen's d	-1.99762
REAC_%	Student's t	1.33754	12.0	0.206	Cohen's d	0.71494
CORT_ACCUM	Student's t	-5.14354[a]	12.0	<.001	Cohen's d	-2.74934
RECOVERY	Student's t	0.00227	12.0	0.998	Cohen's d	0.00121
RECOV_%	Student's t	2.19864	12.0	0.048	Cohen's d	1.17522
AUC	Student's t	-5.55036[a]	12.0	<.001	Cohen's d	-2.96679
AUC_BL	Student's t	-4.79259	12.0	<.001	Cohen's d	-2.56175
CCL4	Student's t	0.96423	12.0	0.354	Cohen's d	0.51540
TNFa	Student's t	0.38692	12.0	0.706	Cohen's d	0.20682
IP-10	Student's t	0.69571	12.0	0.500	Cohen's d	0.37187
CCL4_Change	Student's t	-1.80433	12.0	0.096	Cohen's d	-0.96445
IP10_Change	Student's t	-1.20072	12.0	0.253	Cohen's d	-0.64181
Rest	Student's t	1.17125	12.0	0.264	Cohen's d	0.62606
Move	Student's t	-1.39958[a]	12.0	0.187	Cohen's d	-0.74811
Eat	Student's t	0.51944	12.0	0.613	Cohen's d	0.27765
Jump	Student's t	-1.00614[a]	12.0	0.334	Cohen's d	-0.53781
Hang	Student's t	-0.73063[a]	12.0	0.479	Cohen's d	-0.39054
Sit w/o	Student's t	-0.50711	12.0	0.621	Cohen's d	-0.27106
Sit w/a	Student's t	0.99158	12.0	0.341	Cohen's d	0.53002
1	Student's t	-1.15470	12.0	0.271	Cohen's d	-0.61721
2	Student's t	3.36576	12.0	0.006	Cohen's d	1.79908
3	Student's t	-1.49785	12.0	0.160	Cohen's d	-0.80063
4	Student's t	-0.26746	12.0	0.794	Cohen's d	-0.14296
Tr	Student's t	-2.69347[a]	12.0	0.020	Cohen's d	-1.43972
Tensed	Student's t	0.17695	11.0	0.863	Cohen's d	0.09845
Relaxed	Student's t	0.05570	11.0	0.957	Cohen's d	0.03099
OV	Student's t	-0.69319[a]	11.0	0.503	Cohen's d	-0.38566
Far	Student's t	1.86646	12.0	0.087	Cohen's d	0.99766
Near	Student's t	-0.33464	12.0	0.744	Cohen's d	-0.17887

Independent Samples T-Test by HPA Reactor

		Statistic	df	p		Effect Size
Contact	Student's t	-2.57119	12.0	0.024	Cohen's d	-1.37436
Approach	Student's t	-0.60418	12.0	0.557	Cohen's d	-0.32295
Approach latency (s)	Student's t	-0.29214	12.0	0.775	Cohen's d	-0.15616
Leave	Student's t	-3.14803[a]	12.0	0.008	Cohen's d	-1.68269
Leave Latency (s)	Student's t	2.84542	12.0	0.015	Cohen's d	1.52094
A to L Ratio	Student's t	1.13382	12.0	0.279	Cohen's d	0.60605
Receive Groom	Student's t	-2.67809[a]	12.0	0.020	Cohen's d	-1.43150
Receive Groom (s)	Student's t	-2.65736[a]	12.0	0.021	Cohen's d	-1.42042
Give Groom	Student's t	1.06066[a]	12.0	0.310	Cohen's d	0.56695
Give Groom (s)	Student's t	1.30326[a]	12.0	0.217	Cohen's d	0.69662
Mate	Student's t	0.20316[a]	12.0	0.842	Cohen's d	0.10859
Self-Groom	Student's t	0.17486	12.0	0.864	Cohen's d	0.09347
SM	Student's t	-1.60023[a]	12.0	0.136	Cohen's d	-0.85536
GD	Student's t	0.69706	12.0	0.499	Cohen's d	0.37259
Rest (2)	Student's t	-0.60049	12.0	0.559	Cohen's d	-0.32097
Move (2)	Student's t	0.54509	12.0	0.596	Cohen's d	0.29137
Eat (2)	Student's t	0.58897	12.0	0.567	Cohen's d	0.31482
Jump (2)	Student's t	0.34092	12.0	0.739	Cohen's d	0.18223
Sit	Student's t	1.12989	12.0	0.281	Cohen's d	0.60395
Stand	Student's t	-1.01470	12.0	0.330	Cohen's d	-0.54238
Tr (2)	Student's t	-1.07211	12.0	0.305	Cohen's d	-0.57307
Tensed (2)	Student's t	-0.88280	11.0	0.396	Cohen's d	-0.49115
Relaxed (2)	Student's t	0.85150	11.0	0.413	Cohen's d	0.47373
Head Scans	Student's t	-0.55081	12.0	0.592	Cohen's d	-0.29442
SM (2)	Student's t	0.49605	12.0	0.629	Cohen's d	0.26515
Self Groom	Student's t	1.18032[a]	12.0	0.261	Cohen's d	0.63091
In Bucket	Student's t	0.44699	12.0	0.663	Cohen's d	0.23893
Shrill	Student's t	-1.05466	12.0	0.312	Cohen's d	-0.56374
BCS	Student's t	0.00000	12.0	1.000	Cohen's d	0.00000
Weight	Student's t	0.29632	12.0	0.772	Cohen's d	0.15839
Weight Proj St	Student's t	0.63456	12.0	0.538	Cohen's d	0.33919
Weight 6 months out	Student's t	-0.57935	12.0	0.573	Cohen's d	-0.30968
6 M Weight Chg %	Student's t	-2.30005	12.0	0.040	Cohen's d	-1.22943
Weight Max % Chg Prev Yr %	Student's t	-0.59402	12.0	0.564	Cohen's d	-0.31752
A/G RATIO (CALC)	Student's t	1.40892	12.0	0.184	Cohen's d	0.75310
ALBUMIN	Student's t	1.90888	12.0	0.080	Cohen's d	1.02034
ALK PHOS	Student's t	-0.60921	12.0	0.554	Cohen's d	-0.32564
ALT / SGPT	Student's t	0.65513	12.0	0.525	Cohen's d	0.35018
ANION GAP (CALC)	Student's t	0.91773	12.0	0.377	Cohen's d	0.49055
AST / SGOT	Student's t	1.67286[a]	12.0	0.120	Cohen's d	0.89418
BUN	Student's t	0.62488	12.0	0.544	Cohen's d	0.33401

Independent Samples T-Test by HPA Reactor

		Statistic	df	p		Effect Size
BUN/CREAT RATIO	Student's t	-1.62660	12.0	0.130	Cohen's d	-0.86945
CALCIUM	Student's t	2.26158	12.0	0.043	Cohen's d	1.20886
CARBON DIOXIDE	Student's t	-0.91304	12.0	0.379	Cohen's d	-0.48804
CHLORIDE	Student's t	-0.52810	12.0	0.607	Cohen's d	-0.28228
CHOLESTEROL	Student's t	0.67922	12.0	0.510	Cohen's d	0.36306
CPK	Student's t	0.44520	11.0	0.665	Cohen's d	0.24769
CREATININE	Student's t	1.35213	12.0	0.201	Cohen's d	0.72274
GGT	Student's t	1.90354[a]	11.0	0.083	Cohen's d	1.05903
GLOBULIN (CALC)	Student's t	0.06047	12.0	0.953	Cohen's d	0.03232
GLUCOSE	Student's t	0.32864	12.0	0.748	Cohen's d	0.17567
LDH	Student's t	0.55058[a]	11.0	0.593	Cohen's d	0.30631
PHOSPHORUS	Student's t	1.41043	12.0	0.184	Cohen's d	0.75391
POTASSIUM	Student's t	-0.69283	12.0	0.502	Cohen's d	-0.37033
SODIUM	Student's t	0.10464	12.0	0.918	Cohen's d	0.05593
TOTAL BILIRUBIN	Student's t	0.00000	12.0	1.000	Cohen's d	0.00000
TOTAL PROTEIN	Student's t	1.25280	12.0	0.234	Cohen's d	0.66965
TRIGLYCERIDES	Student's t	0.98829	11.0	0.344	Cohen's d	0.54983
BASO #	Student's t	0.61237	12.0	0.552	Cohen's d	0.32733
BASO %	Student's t	0.72606	12.0	0.482	Cohen's d	0.38810
EOS #	Student's t	-1.47029	12.0	0.167	Cohen's d	-0.78591
EOS %	Student's t	-1.72378	12.0	0.110	Cohen's d	-0.92140
Hematocrit	Student's t	-1.14985[a]	12.0	0.273	Cohen's d	-0.61462
HEMOGLOBIN	Student's t	-1.31629[a]	12.0	0.213	Cohen's d	-0.70359
LYMPH #	Student's t	0.93722	12.0	0.367	Cohen's d	0.50097
LYMPH %	Student's t	1.03712	12.0	0.320	Cohen's d	0.55437
MCH	Student's t	-0.83805	12.0	0.418	Cohen's d	-0.44795
MCHC	Student's t	-0.36452	12.0	0.722	Cohen's d	-0.19484
MCV	Student's t	-0.50663	12.0	0.622	Cohen's d	-0.27081
MONO #	Student's t	-0.96225	12.0	0.355	Cohen's d	-0.51434
MONO %	Student's t	-1.61378	12.0	0.133	Cohen's d	-0.86260
MPV	Student's t	0.52281	12.0	0.611	Cohen's d	0.27945
NEUT #	Student's t	-1.14112	12.0	0.276	Cohen's d	-0.60995
NEUT %	Student's t	-0.89371	12.0	0.389	Cohen's d	-0.47771
NRBC %	Student's t	1.22429[a]	12.0	0.244	Cohen's d	0.65441
PLATELET COUNT	Student's t	0.22713	12.0	0.824	Cohen's d	0.12141
RBC	Student's t	-1.10116[a]	12.0	0.292	Cohen's d	-0.58859
RDW	Student's t	0.51489	12.0	0.616	Cohen's d	0.27522
WBC	Student's t	0.41831	12.0	0.683	Cohen's d	0.22360
A/G RATIO (CALC) (2)	Student's t	-0.49692	11.0	0.629	Cohen's d	-0.27646
ALBUMIN (2)	Student's t	1.28677	11.0	0.225	Cohen's d	0.71589
ALK PHOS (2)	Student's t	-0.41520	11.0	0.686	Cohen's d	-0.23100

Independent Samples T-Test by HPA Reactor

		Statistic	df	p		Effect Size
ALT / SGPT (2)	Student's t	-0.18415	11.0	0.857	Cohen's d	-0.10245
ANION GAP (CALC) (2)	Student's t	0.07528	11.0	0.941	Cohen's d	0.04188
AST / SGOT (2)	Student's t	-0.70026[a]	11.0	0.498	Cohen's d	-0.38959
BUN (2)	Student's t	-0.30105	11.0	0.769	Cohen's d	-0.16749
BUN/CREAT RATIO (2)	Student's t	-1.60244	11.0	0.137	Cohen's d	-0.89151
CALCIUM (2)	Student's t	2.24986	11.0	0.046	Cohen's d	1.25171
CARBON DIOXIDE (2)	Student's t	0.53243	11.0	0.605	Cohen's d	0.29622
CHLORIDE (2)	Student's t	0.43019	11.0	0.675	Cohen's d	0.23933
CHOLESTEROL (2)	Student's t	-0.82192[a]	11.0	0.429	Cohen's d	-0.45727
CPK (2)	Student's t	-0.15100	11.0	0.883	Cohen's d	-0.08401
CREATININE (2)	Student's t	1.03304	11.0	0.324	Cohen's d	0.57473
GGT (2)	Student's t	0.36243	11.0	0.724	Cohen's d	0.20164
GLOBULIN (CALC) (2)	Student's t	1.24484	11.0	0.239	Cohen's d	0.69257
GLUCOSE (2)	Student's t	-1.90050	11.0	0.084	Cohen's d	-1.05734
LDH (2)	Student's t	0.42608	11.0	0.678	Cohen's d	0.23705
PHOSPHORUS (2)	Student's t	2.18152	11.0	0.052	Cohen's d	1.21369
POTASSIUM (2)	Student's t	1.71325	11.0	0.115	Cohen's d	0.95317
SODIUM (2)	Student's t	0.69775	11.0	0.500	Cohen's d	0.38819
TOTAL BILIRUBIN (2)	Student's t	1.12349	11.0	0.285	Cohen's d	0.62505
TOTAL PROTEIN (2)	Student's t	1.66853	11.0	0.123	Cohen's d	0.92828
TRIGLYCERIDES (2)	Student's t	0.58422	11.0	0.571	Cohen's d	0.32503
BASO # (2)	Student's t	-0.50383	11.0	0.624	Cohen's d	-0.28031
BASO % (2)	Student's t	-0.99871	11.0	0.339	Cohen's d	-0.55563
EOS # (2)	Student's t	0.69494	11.0	0.502	Cohen's d	0.38663
EOS % (2)	Student's t	0.27794	11.0	0.786	Cohen's d	0.15463
Hematocrit (2)	Student's t	0.27235	11.0	0.790	Cohen's d	0.15152
HEMOGLOBIN (2)	Student's t	-0.05042	11.0	0.961	Cohen's d	-0.02805
LYMPH # (2)	Student's t	1.75457[a]	11.0	0.107	Cohen's d	0.97615
LYMPH % (2)	Student's t	-0.22516	11.0	0.826	Cohen's d	-0.12527
MCH (2)	Student's t	-1.18334	11.0	0.262	Cohen's d	-0.65835
MCHC (2)	Student's t	-1.38661	11.0	0.193	Cohen's d	-0.77144
MCV (2)	Student's t	-0.49230	11.0	0.632	Cohen's d	-0.27389
MONO # (2)	Student's t	-1.60e−17	11.0	1.000	Cohen's d	-8.92e−18
MONO % (2)	Student's t	-1.55871	11.0	0.147	Cohen's d	-0.86719
MPV (2)	Student's t	0.94122	11.0	0.367	Cohen's d	0.52365
NEUT # (2)	Student's t	1.85130	11.0	0.091	Cohen's d	1.02997
NEUT % (2)	Student's t	0.92415	11.0	0.375	Cohen's d	0.51415
NRBC % (2)	Student's t	-0.56076	11.0	0.586	Cohen's d	-0.31198
PLATELET COUNT (2)	Student's t	-0.18243	11.0	0.859	Cohen's d	-0.10149
RBC (2)	Student's t	0.34476	11.0	0.737	Cohen's d	0.19181
RDW (2)	Student's t	0.51327	11.0	0.618	Cohen's d	0.28556

Independent Samples T-Test by HPA Reactor

		Statistic	df	p		Effect Size
WBC (2)	Student's t	2.20297[a]	11.0	0.050	Cohen's d	1.22562

Note. H_a $\mu_{NR} \neq \mu_{Reactive}$

[a] Levene's test is significant ($p < .05$), suggesting a violation of the assumption of equal variances

SI Table 3. T-Tests for each measured parameter based on HPA reactor. Behavioral variables followed by “(2)” are isolation behaviors. Blood CBC/Chem parameters are averaged or parameters followed by “(2)” are change from post-pre. Positive t-statistics indicate non-reactor > reactor. For clarification on Behavioral variable names, see Table 1.
